# Sex differences in metabolic regulation and diabetes susceptibility

**DOI:** 10.1007/s00125-019-05040-3

**Published:** 2019-11-21

**Authors:** Blandine Tramunt, Sarra Smati, Naia Grandgeorge, Françoise Lenfant, Jean-François Arnal, Alexandra Montagner, Pierre Gourdy

**Affiliations:** 1 Institut des Maladies Métaboliques et Cardiovasculaires (I2MC), UMR1048, Team 9, INSERM/UPS, Université de Toulouse, 1 avenue Jean Poulhès, BP 84225, 31432 Toulouse Cedex 4, France; 2 Service de Diabétologie, Maladies Métaboliques et Nutrition, CHU de Toulouse, Toulouse, France; 3 Institut National de la Recherche Agronomique (INRA), Toxalim UMR 1331, Toulouse, France

**Keywords:** Diabetes, Energy balance, Glucose metabolism, Oestrogens, Review, Sex differences

## Abstract

Gender and biological sex impact the pathogenesis of numerous diseases, including metabolic disorders such as diabetes. In most parts of the world, diabetes is more prevalent in men than in women, especially in middle-aged populations. In line with this, considering almost all animal models, males are more likely to develop obesity, insulin resistance and hyperglycaemia than females in response to nutritional challenges. As summarised in this review, it is now obvious that many aspects of energy balance and glucose metabolism are regulated differently in males and females and influence their predisposition to type 2 diabetes. During their reproductive life, women exhibit specificities in energy partitioning as compared with men, with carbohydrate and lipid utilisation as fuel sources that favour energy storage in subcutaneous adipose tissues and preserve them from visceral and ectopic fat accumulation. Insulin sensitivity is higher in women, who are also characterised by higher capacities for insulin secretion and incretin responses than men; although, these sex advantages all disappear when glucose tolerance deteriorates towards diabetes. Clinical and experimental observations evidence the protective actions of endogenous oestrogens, mainly through oestrogen receptor α activation in various tissues, including the brain, the liver, skeletal muscle, adipose tissue and pancreatic beta cells. However, beside sex steroids, underlying mechanisms need to be further investigated, especially the role of sex chromosomes, fetal/neonatal programming and epigenetic modifications. On the path to precision medicine, further deciphering sex-specific traits in energy balance and glucose homeostasis is indeed a priority topic to optimise individual approaches in type 2 diabetes prevention and treatment.

**Electronic supplementary material:**

The online version of this article (10.1007/s00125-019-05040-3) contains a slideset of the figures for download, which is available to authorised users.

## Introduction

In the last few years, addressing gender and sex differences has emerged as a priority topic in several medical areas, including metabolic diseases [1]. While gender mainly refers to the socially constructed identities of individuals, sex dimorphism relies on the fundamental biological disparities that differently influence physiological or pathophysiological processes in males and females. Although not fully understood, underlying mechanisms largely involve sex steroid hormones and sex chromosomes but also include sex specificities in fetal/neonatal programming and epigenetic modifications. Recent guidelines, thus, emphasise the need to consider such sex differences during preclinical (cellular and animal models) to clinical research efforts, avoiding the traditional male predominance when using these approaches [2].

It is now obvious that sex has a significant impact on the pathogenesis of metabolic disorders, such as type 2 diabetes. The first dimorphic aspect concerns diabetes prevalence, with a male predominance reported in humans and also in most animal models, with females being generally protected from diet-induced metabolic disorders [3]. Therefore, the present review aims to discuss how sex differences in energy balance and metabolic homeostasis influence susceptibility to diabetes, with a specific focus on the protective actions of endogenous oestrogens.

## Diabetes is more prevalent in men: epidemiological evidence

Except in some parts of the world, such as the Middle East and North Africa, diabetes is more prevalent in men than in women, especially in middle-aged populations. Analysing 751 population-based studies (4.4 million adults from 146 countries), the NCD Risk Factor Collaboration first showed that age-standardised prevalence rates more markedly increased in men (4% to 9%) than in women (5% to 8%) between 1980 and 2014, despite some substantial disparities across geographical areas [4]. Similarly, the US National Health and Nutrition Examination Survey recently reported a higher prevalence of diabetes among men compared with women (13% vs 11% for the 2013–2016 period, in adults aged 20–79 years) [5]. The last global estimates published by the International Diabetes Federation also indicate sex differences in worldwide diabetes prevalence in adult populations (9.1% in men vs 8.4% in women), suggesting that about 12.3 million more men than women worldwide were living with diabetes in 2017. The peak in diabetes prevalence occurs earlier in men (65–69 years of age) than in women (70–79 years of age) and male predominance is, therefore, specifically observed in middle-aged populations (35–69 years of age) [6].

Studies offering systematic screening procedures in large populations confirmed a male predominance when diagnosis of diabetes was based on fasting plasma glucose (FPG) and/or HbA_1c_ measurements, but not when considering 2 h plasma glucose after an OGTT (2hPG-OGTT). Among the 7680 men and 9251 women included in the European Diabetes Epidemiology: Collaborative analysis Of Diagnostic criteria in Europe (DECODE) study, undiagnosed diabetes and impaired fasting glucose, both defined by isolated FPG, were more prevalent in men in the 30–69 years age range. However, the prevalence of impaired glucose tolerance, was higher in women in all age groups [7]. In 13,016 inhabitants (aged 30–60 years) of the Copenhagen county (Denmark) who participated in the Inter99 study, diagnosis of dysglycaemia was reported in 49.6% (95% CI 43.4%, 55.6%) of men and 34.6% (95% CI 28.6%, 41.0%) of women by the age of 60 years. The risk of diabetes (OR 1.7 [95% CI 1.3, 2.1]) and impaired fasting glucose (OR 3.0 [95% CI 2.4, 3.7]), but not of impaired glucose tolerance (OR 1.0 [95% CI 0.9, 1.2]), appeared to be higher in men than in women [8]. In individuals with normal glucose tolerance, women generally exhibit lower FPG and HbA_1c_ levels but increased 2hPG-OGTT levels, as compared with men [9, 10]. However, these differences could be the consequence of challenging all individuals with the same amount of glucose, regardless of sex-dependent characteristics, such as body size, muscle mass or physical fitness [9], but they could also be owing to delayed gut glucose absorption in women as compared with men [11]. These later observations perfectly illustrate the need for considering both sexes, as well as their phenotypic and biological specificities, in all studies devoted to metabolic regulation.

## A critical role for sex steroid hormones in diabetes susceptibility

Both clinical and experimental studies indicate that post-pubertal sex steroid hormones largely contribute to sex differences in diabetes susceptibility. The protective role of endogenous oestrogens in women is evidenced by the deleterious impact of the menopause on body composition and glucose homeostasis, leading to an increased incidence of metabolic disorders vs premenopausal women [12]. Early menopause and premature ovarian insufficiency are associated with an increased risk of type 2 diabetes as compared with premenopausal women, while a 21–35% reduction in diabetes incidence has been reported in menopausal women receiving oestrogen-based hormonal therapy vs placebo [13–15]. Further demonstrating the contribution of the oestrogen pathway to diabetes susceptibility in humans, rare loss-of-function mutations in the gene encoding either aromatase (the enzyme that converts androgens into oestrogens) or oestrogen receptor α (ERα) result in dysmetabolic phenotypes in individuals of both sexes [16]. Similarly, deletion of aromatase or ERα in transgenic mice also leads to obesity, insulin resistance and impaired glucose tolerance [17, 18]. Moreover, in all animal models, oestrogen-associated protection of females from high-fat-diet (HFD)-induced obesity and hyperglycaemia is totally abolished by bilateral ovariectomy, but restored by oestrogen administration [18, 19].

Androgens are also associated with metabolic risks, but mainly in pathophysiological situations leading to unbalanced androgen/oestrogen ratio. In men with hypogonadism, low testosterone plasma concentrations are correlated with an increased risk of type 2 diabetes and vascular diseases, while testosterone supplementation clearly improves glucose and lipid homeostasis [20]. However, besides the direct activation of androgen receptors, part of the metabolic actions of testosterone can also result from indirect mechanisms, through its aromatisation into oestrogens. Conversely, androgen excess can lead to significant metabolic alterations in women. High testosterone plasma levels are thought to favour insulin resistance and diabetes in women with polycystic ovary syndrome (PCOS), but the most direct demonstration comes from the development of metabolic disorders in transsexual people on high-dose androgens [3]. Furthermore, dihydrotestosterone administration was recently reported to predispose female mice to diabetes by promoting insulin resistance and beta cell failure through androgen receptor activation in neurons and beta cells, respectively [21]. Overall, it is now clear that androgens play a complex role in the pathogenesis of obesity and type 2 diabetes in both males and females, as recently reviewed [21, 22].

**Fig. 1:**
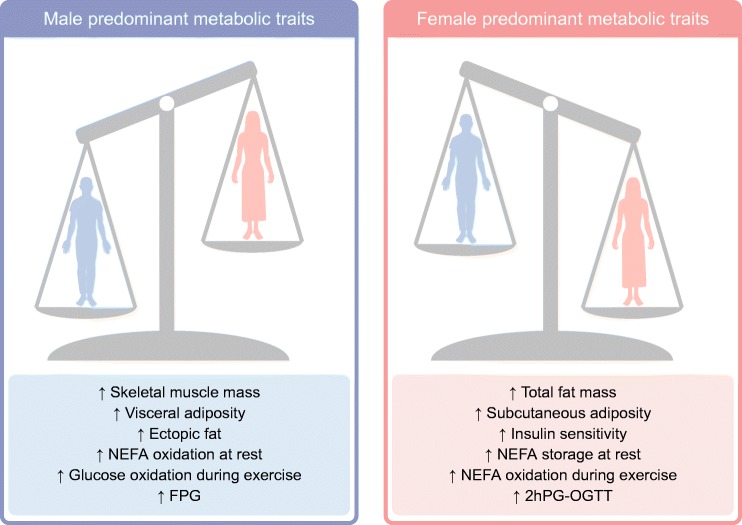
Main sex dimorphisms in body composition and metabolic homeostasis in humans (premenopausal women vs age-matched men). This figure is available as part of a downloadable slideset

**Fig. 2:**
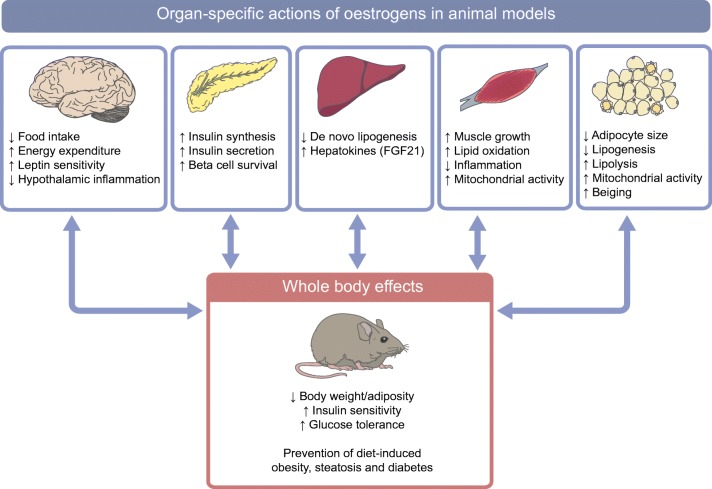
Tissue-specific actions of oestrogens on energy balance and metabolic regulation in rodent models. FGF21, fibroblast growth factor 21. This figure is available as part of a downloadable slideset

In summary, although the androgen/oestrogen ratio undoubtedly has an impact on metabolic regulation, both human and animal studies demonstrate that endogenous oestrogens protect females from type 2 diabetes, at least during their reproductive life. As detailed below, oestrogens largely contribute to sexual dimorphisms in energy balance and metabolic homeostasis, which are the main determinants of sex differences in type 2 diabetes susceptibility. The main sexually dimorphic body composition and metabolic traits in humans, and the tissue-specific actions of oestrogens (as reported in animal models) are summarised in Figs 1 and 2, respectively.

## Biological sex as a determinant of energy balance and body composition

### Sex influences energy partitioning

The sexual dimorphism regarding energy partitioning is classically viewed as an evolutionary adaptation allowing females to better withstand periods of undernutrition, with the ultimate aim of preserving their reproductive functions. Energy storage is generally favoured in females, whereas males predominantly mobilise energy stores to enable sustained physical activity [3]. Sex differences in adipose tissue distribution respond to these physiological considerations, with a predominance of subcutaneous tissue in women, which is better adapted for large and long-term storage. Further supporting functional differences in adipose tissue, sex-specific gene expression signatures were recently found in human abdominal and gluteal subcutaneous depots [23].

Sex also influences the utilisation of carbohydrates and lipids as fuel sources. At rest and during the post-absorptive state, women are more likely to incorporate NEFAs into triacylglycerols, thus promoting fat storage, whereas men are more prone to produce energy through plasma NEFA oxidation. Metabolic adaptation during exercise also differs between the sexes since women preferentially oxidise lipids while men use carbohydrates as the predominant fuel source [3].

Known to play a critical role in the regulation of energy storage and metabolic fluxes, in a functional perspective, the liver is undoubtedly one of the most sexually dimorphic organs [24]. Indeed, in order to regulate fertility in relation to nutrient availability, activation of ERα in hepatocytes adapts hepatic metabolism in female mice to control lipid synthesis, lipoprotein production and IGF-1 expression [24]. Moreover, while male mice restrain lipogenic and gluconeogenic pathways to preserve amino acid reserves in conditions of short-term fasting, female mice maintain hepatic lipid synthesis using amino acids as a fuel source, clearly illustrating sex differences in liver-associated metabolic adaptations [25].

### Sex specificities in energy expenditure

Contrasting with observations in rodent models, clear differences have not been demonstrated in energy expenditure between women and men when adjusted for lean mass [26]. The relative contribution of fat mass to resting metabolic rate is higher in women than in men, even after adjustment for plasma sex steroid concentrations, body fat distribution and insulin sensitivity [27]. This female trait correlates with higher expression of genes involved in mitochondrial function in subcutaneous adipose tissues, including *UCP1* [27]. Accordingly, upon the recent study of sex differences in cardio-metabolic traits in a large panel of inbred mouse strains, males were found to have reduced mitochondrial function in adipose tissues, which was associated with an increased susceptibility to obesity and metabolic disorders. These sex differences correlated with the expression of a cluster of genes involved in adipose tissue ‘beiging’ and mitochondrial functions in adipose tissues [28]. In line with this observation, oestrogens have recently been demonstrated to enhance thermogenesis in brown adipose tissue and to promote beiging of white adipocytes. Indeed, in vitro and in vivo approaches have demonstrated that selective activation of ERα induces adipose tissue beiging through induction of AMP-activated protein kinase (AMPK) and adipose triglyceride lipase (ATGL)-mediated lipolysis, resulting in NEFA generation and uncoupling protein 1 (UCP-1) activation [29]. Finally, as revealed by ^18^F-fluoro-2-deoxy-d-glucose (^18^F-FDG) positron-emission tomography–computed tomography (PET–CT) scanning, brown adipose tissue is better preserved and more metabolically active in women than in men, thus contributing to enhanced energy expenditure in the former [30, 31].

Besides their influence on adipose tissues, oestrogens contribute to sexual dimorphism in energy balance through direct effects on the central nervous system (CNS). In rodent models, ERα activation in hypothalamic pro-opiomelanocortin (POMC) and steroidogenic factor-1 (SF-1) neurons controls food intake and energy expenditure, respectively [32]. More specifically, ERα signalling induces AMPK inhibition in the ventromedial nucleus, leading to enhanced thermogenesis in brown adipose tissue through the sympathetic nervous system [33]. Oestrogens also enhance leptin sensitivity within the brain, reinforcing their impact on food intake [34]. In addition, as compared with males, female mice are less prone to HFD-induced hypothalamic inflammation and lipotoxicity; in the CNS they have lower concentrations of saturated fatty acids and sphingolipids but higher amounts of polyunsaturated fatty acids [35].

In contrast, oestrogen-induced peripheral signals are also able to regulate energy expenditure. For instance, ERα activation in hepatocytes indirectly promotes energy expenditure in female mice by enhancing fibroblast growth factor 21 (FGF21) synthesis, thus conferring protection against adipose tissue accumulation [36].

### Consequences on body composition and ectopic fat

As compared with age-matched men, healthy premenopausal women exhibit higher global fat mass and reduced fat-free mass due to lower skeletal muscle mass. As previously mentioned, women are characterised by an increased propensity to store adipose tissue in subcutaneous sites, especially in gluteofemoral locations, as compared with preferential deposition in visceral area in men. This leads to significant sex differences in body composition [3, 26]. Of note, women are also less susceptible to ectopic fat deposition in most tissues, such as the liver or the myocardium. Women are, thus, protected from non-alcoholic fatty liver disease (NAFLD) before menopause, with the protective role of oestrogens having been evidenced experimentally [37]. Consistent with this, lower dietary fatty acid oxidation and a sustained increase in de novo lipogenesis in the liver have been reported in healthy men, as compared with women [38]. Conversely, women have a higher propensity to accumulate intramyocellular lipids in leg skeletal muscles, but without deleterious consequences on insulin sensitivity [39]. This probably explains why, despite a female predominance in the worldwide prevalence of obesity, diabetes is more prevalent in men [3]. Interestingly, at least in middle-aged populations of European origin, women have a higher BMI than men at diagnosis of type 2 diabetes [40].

Although ageing induces body composition changes in both sexes, menopause triggers the progressive accumulation of visceral fat that contributes to the increased risk of metabolic disorders [12]. Sex steroids influence body composition in both sexes and post-menopausal changes, thus, illustrates the beneficial role of oestrogens in women. Recent data from mouse models also reveal that oestrogen signalling in adipocytes protects mice from adipose tissue inflammation and fibrosis and, thus, contributes to the prevention of obesity [41]. However, sex steroids are not the only contributors to the sexual dimorphism in body composition. Indeed, new mouse models that allow us to dissociate the specific contribution of sex chromosomes from the influence of gonadal hormones have recently provided evidence that the number of X chromosomes is positively associated with weight gain and adiposity, whereas the Y chromosome exerts deleterious effects on glucose metabolism [42].

Finally, it is obvious that such sex-specific biological traits interfere with environmental determinants to modulate individual susceptibility to obesity and type 2 diabetes in humans. For instance, gender-specific patterns in dietary behaviour, mainly influenced by cultural and social factors, are likely to have an impact on the incidence of metabolic disorders in both sexes [43].

## Sex-dimorphic traits in the regulation of glucose homeostasis

### Females are more insulin sensitive than males

In a large population of individuals with normal blood glucose levels, insulin sensitivity was assessed with the oral glucose insulin sensitivity index and found to be higher in women than in men, even after adjustment for age and BMI [10]. However, this sex advantage disappears when glucose tolerance deteriorates towards type 2 diabetes, with a similar extent of insulin resistance observed in both sexes [44]. Hyperinsulinaemic−euglycaemic clamp studies confirm that healthy women are more sensitive to insulin than men when matched for physical fitness (41% increase in whole body insulin sensitivity). This is due to enhanced glucose uptake by skeletal muscle in women [45, 46]. In agreement with this, sex differences have been reported in muscle characteristics, with a higher proportion of type I fibres and capillary density in women, which both favour enhanced insulin action [46].

The observation that women are less prone to insulin resistance than men is rather unexpected considering their increased fat mass, circulating NEFA levels and lipid content in myocytes, as well as a lower skeletal muscle mass. As a plausible explanation, experimental data indicate that women are protected from NEFA-induced insulin resistance and, thus, more resistant to lipotoxicity, especially in skeletal muscles [47]. Oestrogens confer protection against insulin resistance through activation of the ERα pathway in insulin-sensitive tissues, as demonstrated in mouse models [18, 19]. For example, in mice with specific myocyte ERα deletion, muscle-associated oxidative metabolism was altered and hyperglycaemia developed, indicating that oestrogens preserve mitochondrial function and insulin sensitivity [48]. The liver is also involved in this phenomenon, since ERα signalling in hepatocytes mediates protective effects against steatosis and insulin resistance in HFD-fed female mice [49].

### Sex also has an impact on pancreatic endocrine function

In normoglycaemic individuals, estimations of beta cell function following an OGTT or a standardised meal suggest that women exhibit a greater insulin secretion capacity than men [10]. Insulin secretion is more markedly increased in obese men, as a way to compensate for a higher level of insulin resistance. However, type 2 diabetes is characterised by similar impairments in beta cell function in both sexes [44]. Besides functional differences, analysis of pancreatic biopsies from human donors recently estimated that islets from women contain 6% more beta cells than those from men [50].

Using human islets or rodent models, experimental studies demonstrate that sex hormones act as key regulators of islet biology in a sex-specific manner. More specifically, endogenous oestrogens stimulate insulin synthesis and secretion and exert protective effects on islets from females, preserving beta cell function and preventing apoptosis induced by metabolic injuries, such as oxidative stress and lipotoxicity [51]. Interestingly, the beneficial actions of oestrogens on beta cells could explain why the male predominance in diabetes prevalence is not restricted to type 2 diabetes but also applies to insulin-deficient forms of diabetes, such as type 1 diabetes, that are diagnosed post puberty [52]. Indeed, type 1 diabetes incidence is characterised by a sex ratio close to 1 in children, but a significant male excess (sex ratio ~1.7) is reported in the 15–40 year age range, mainly in populations of European origin [53].

Finally, it has been proposed that enhanced insulin secretion in women could also reflect sex differences in glucose-dependent glucagon-like peptide-1 (GLP-1) secretion. Normoglycaemic women were, indeed, characterised by a 20% increase in serum GLP-1 concentrations following an OGTT as compared with men, even after adjustment for BMI. This sex difference was no longer observed in individuals with prediabetes or type 2 diabetes, irrespective of age or weight [54]. Further supporting the enhancing effect of oestrogens on incretin response, oestradiol was demonstrated to positively regulate proglucagon-derived peptide secretion in mouse and human alpha and L cells [55].

## Future directions: how far are we from a sex-specific medicine in diabetes?

Alongside the critical roles of oestrogens (as described in this review), the complex mechanisms responsible for sex-dimorphic metabolic regulation need to be further characterised. It is suggested that analysis of sex steroid balance in males and females cannot be restricted to circulating hormones levels but should also integrate their molecular regulation at the tissue level. For example, aromatisation should be further considered, especially in well-recognised sites of oestrogen biosynthesis, such as the brain or adipose tissues. In addition, it is also important to consider the regulation of local steroid activity resulting from sulfonation and desulfation processes, which lead to hormonally deactivated or activated molecules, respectively. In mouse models, both steroid sulfatase and oestrogen sulfotransferase (EST) have been demonstrated to interfere with the pathogenesis of type 2 diabetes in a sex-specific manner [56]. For instance, inactivation of EST, the enzyme responsible for oestrogen deactivation, increases energy expenditure, improves insulin sensitivity, and reduces hepatic gluconeogenesis and lipogenesis in different mouse models of obesity-related metabolic disorders, but only in females [57]. Although not easy to address (and largely underestimated until now), such fine regulation of the paracrine and intracrine actions of sex steroids could be crucial for local metabolic regulation in both sexes.

Interesting new insights have been provided into the contribution of sex chromosomes to dimorphic gene expression in metabolic tissues [58]. New fields are also currently being explored, such as the role of the gut microbiome in sex-biased susceptibility to metabolic disorders [59]. Finally, in addition to genetic differences, sex-specific epigenetic modifications in responses to various physiological or pathophysiological situations, including exposure to hyperglycaemia and environmental factors, undoubtedly represent an additional layer for integration into the determinants of sex differences in metabolism [3, 60]. Therefore, the study of sexual dimorphism in metabolism should no longer be limited to the period of reproductive life alone, but considered from the preconception period and during the entire life.

Deciphering sex-specific traits in energy balance and glucose homeostasis is certainly of major interest to optimise individual approaches to diabetes prevention and treatment. Sex has already been reported to influence therapeutic responses in type 2 diabetes. For instance, insulin-naive women initiating a basal insulin regimen showed a smaller improvement in HbA_1c_ associated with an increased rate of hypoglycaemia vs men [61]. This may be related to the fact that women exhibit reduced counter-regulatory hormonal response (glucagon and adrenaline [epinephrine]), together with lower rates of endogenous glucose production compared with men [62]. More widely, to definitely consider sex as a pillar of precision medicine, sex dimorphisms in metabolic pathways still need to be better characterised in humans, considering the effect of age, ethnic origin and pathophysiological status, such as the different phenotypical clusters of diabetes.

Finally, studying sex differences in metabolism could also lead to the development of new therapeutic approaches targeting sex-dimorphic metabolic pathways or sex hormone receptors. Countering the deleterious metabolic effects of menopause in women at risk of type 2 diabetes is obviously a priority objective in terms of public health. Beyond lifestyle adaptations, hormone replacement therapy has been associated with reduced type 2 diabetes incidence in clinical trials, as previously mentioned [14, 15], but the uncertainties regarding its benefit–risk balance do not allow for its extended use in this context. Menopausal women could, thus, particularly benefit from new selective oestrogen receptor modulators that are able to mediate the protective actions of oestrogens on body composition and glucose metabolism with limited side effects on reproductive tissues [63]. Tissue-specific targeting could also be a relevant strategy, as illustrated by the protection conferred by a GLP-1–oestrogen conjugate against diet-induced obesity and glucose intolerance in mice via selective ERα activation in the CNS and the pancreas [64].

## Conclusion

It is now clear that many aspects of energy and glucose homeostasis are regulated differently in males and females, influencing their predisposition to diabetes and associated metabolic disorders. Moreover, sex biases have also been described in the occurrence and the progression of diabetic complications, reinforcing the need for sex-specific approaches in diabetes management [65]. As in almost all diseases, personalised management of diabetes should take into account the sex of the patient.

## Electronic supplementary material

Slideset of figures (PPTX 278 kb)

## Abbreviations

AMPK: AMP-activated protein kinase
CNS: Central nervous system
ERα: Oestrogen receptor α
EST: Oestrogen sulfotransferase
FPG: Fasting plasma glucose
GLP-1: Glucagon-like peptide-1
HFD: High-fat-diet
2hPG-OGTT: 2 h plasma glucose after an OGTT
