# Supplementary material for: Sex differences in metabolic regulation and diabetes susceptibility
Source: Diabetologia. 2019 Nov 21;63(3):453–61. doi: 10.1007/s00125-019-05040-3 (PMC6997275; doi:10.1007/s00125-019-05040-3)
Supplement: Supplementary file 1 — (PPTX 278 kb) [file 125_2019_5040_MOESM1_ESM.pptx]

## Slide 1
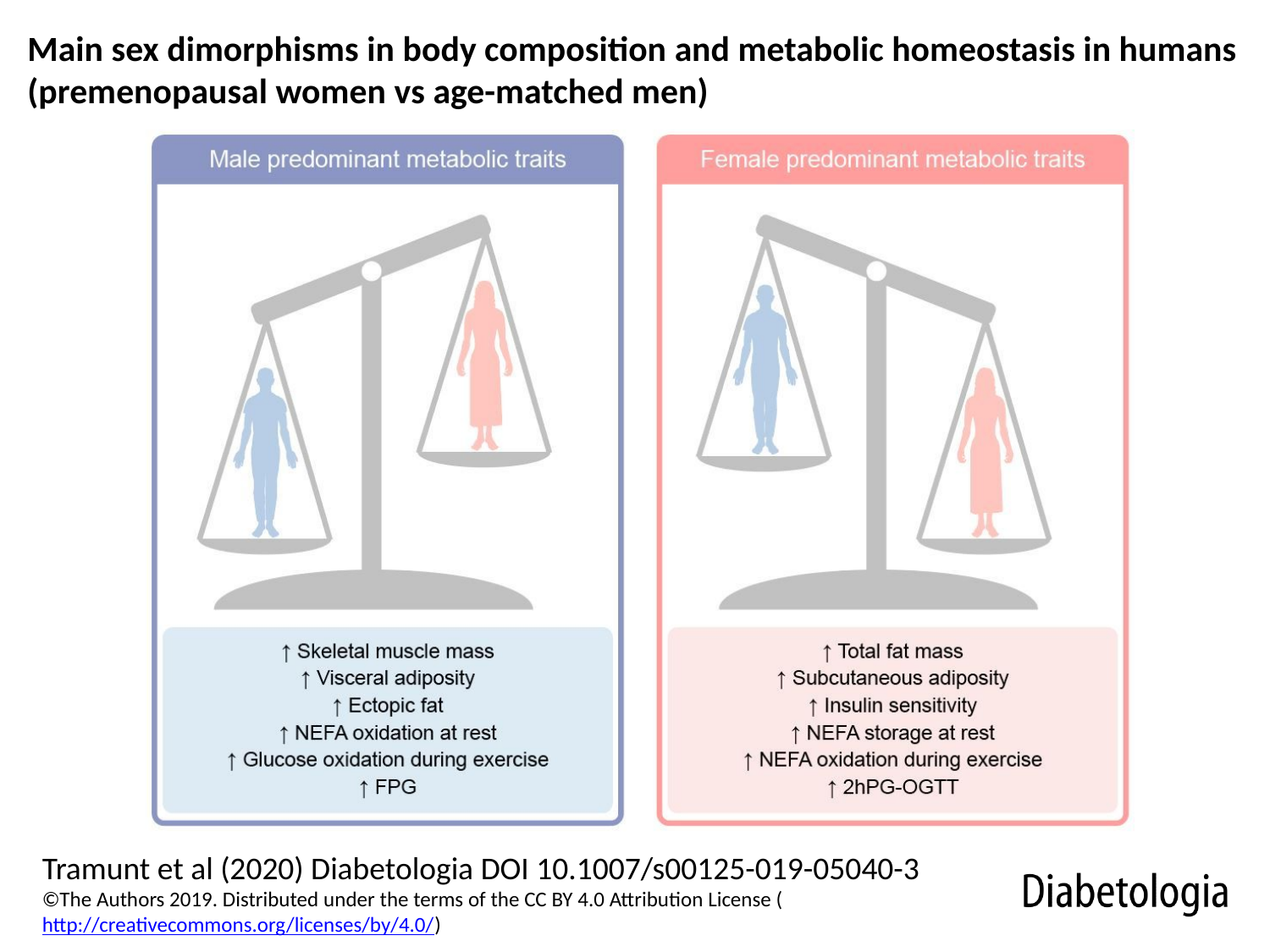

Main sex dimorphisms in body composition and metabolic homeostasis in humans (premenopausal women vs age-matched men)
Tramunt et al (2020) Diabetologia DOI 10.1007/s00125-019-05040-3
©The Authors 2019. Distributed under the terms of the CC BY 4.0 Attribution License (http://creativecommons.org/licenses/by/4.0/)

## Slide 2
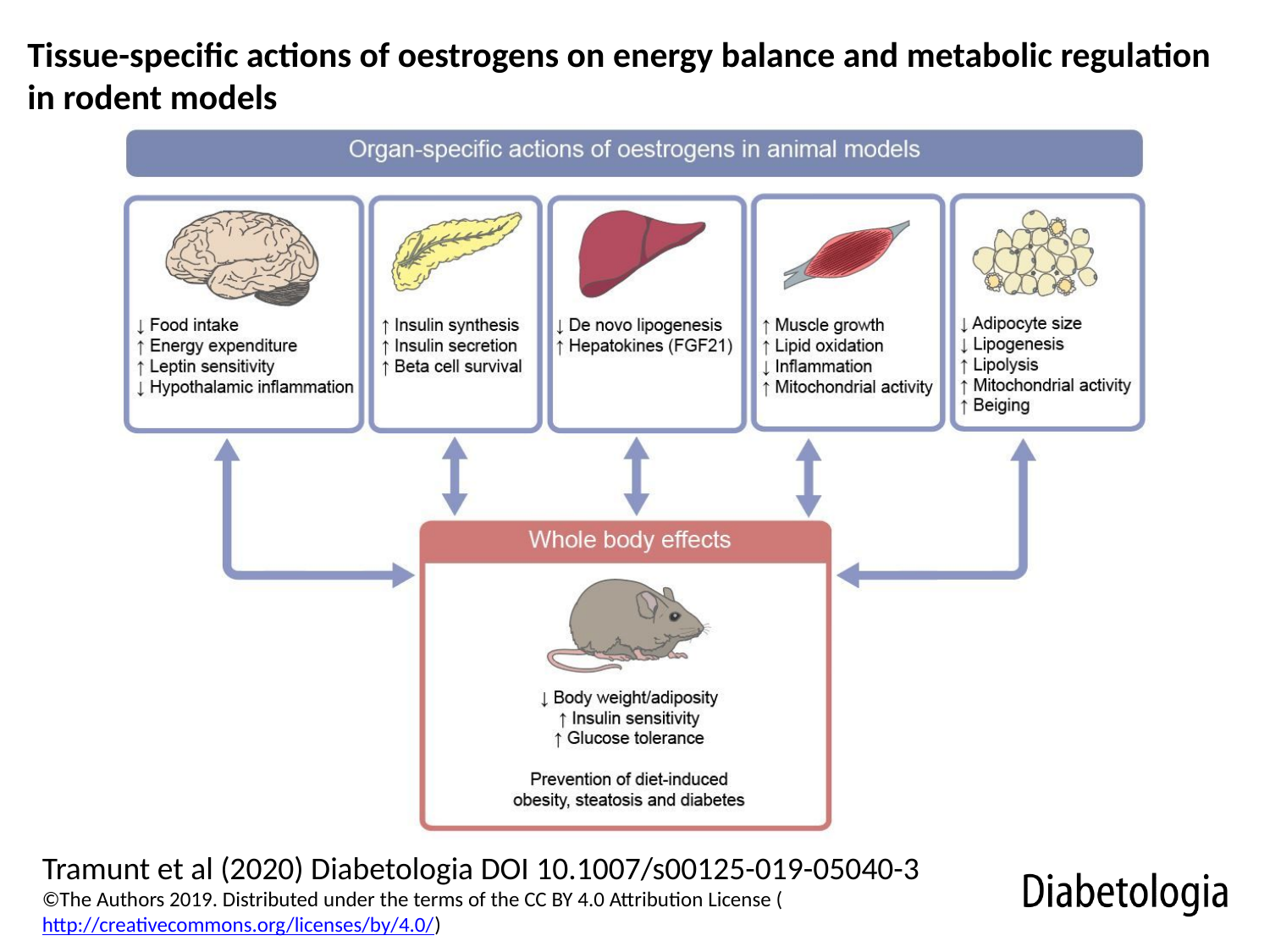

Tissue-specific actions of oestrogens on energy balance and metabolic regulation in rodent models
Tramunt et al (2020) Diabetologia DOI 10.1007/s00125-019-05040-3
©The Authors 2019. Distributed under the terms of the CC BY 4.0 Attribution License (http://creativecommons.org/licenses/by/4.0/)
